# Normative reference equations for leg discomfort during incremental cardiopulmonary cycle exercise testing in older adults

**DOI:** 10.1111/cpf.70083

**Published:** 2026-07-23

**Authors:** Rachelle Aucoin, Dennis Jensen, Michael Stickland, Andrew Brotto, Hayley Lewthwaite, Pei Zhi Li, Jean Bourbeau, Wan C. Tan, Magnus Ekström, CanCOLD Collaborative Research Group, Jean Bourbeau, Wan C. Tan, J Mark FitzGerald, Don D. Sin, Darcy D. Marciniuk, Denis E. O'Donnell, Paul Hernandez, Kenneth R. Chapman, Brandie Walker, Shawn Aaron, François Maltais

**Affiliations:** ^1^ Respiratory Epidemiology Team, Faculty of Medicine Dalhousie University Halifax Nova Scotia Canada; ^2^ Clinical Exercise and Respiratory Physiology Laboratory, Department of Kinesiology & Physical Education McGill University Montréal Quebec Canada; ^3^ Translational Research in Respiratory Diseases Program and Respiratory Epidemiology and Clinical Research Unit Research Institute of the McGill University Health Centre Montréal Quebec Canada; ^4^ Division of Pulmonary Medicine, Faculty of Medicine and Dentistry University of Alberta, Edmonton, AB, Canada, G.F. MacDonald Centre for Lung Health, Covenant Health Edmonton Alberta Canada; ^5^ UniSA: Allied Health and Human Performance, Innovation, Implementation and Clinical Translation in Health University of South Australia Adelaide Australia; ^6^ Montreal Chest Institute McGill University Health Center Research Institute, McGill University Québec Montréal Canada; ^7^ Department of Medicine University of British Columbia Centre for Heart Lung Innovation Vancouver British Columbia Canada; ^8^ Faculty of Medicine, Department of Clinical Sciences Lund, Respiratory Medicine, Allergology and Palliative Medicine Lund University Lund Sweden; ^9^ McGill University Montreal Quebec Canada; ^10^ University of British Columbia Vancouver British Columbia Canada; ^11^ University of Saskatoon Saskatoon Saskatchewan Canada; ^12^ Queen's University Kingston Ontario Canada; ^13^ Dalhousie University Halifax Nova Scotia Canada; ^14^ University of Toronto Toronto Ontario Canada; ^15^ University of Calgary Calgary Alberta Canada; ^16^ University of Ottawa Ottawa Ontario Canada; ^17^ University of Laval Quebec City Quebec Canada

**Keywords:** cardiopulmonary exercise testing, expected response, healthy people, leg discomfort, normative reference equations

## Abstract

**Background:**

Leg discomfort, assessed with the Borg category‐ratio 0–10 (Borg CR10) scale, is a primary reason for exercise cessation in both health and disease. However, interpretation during cardiopulmonary exercise testing (CPET) is limited by the absence of normative reference equations.

**Purpose:**

Develop normative reference equations for leg discomfort during CPET in relation to absolute and relative power output (W) and rate of oxygen uptake (V'O_2_).

**Methods:**

This was a retrospective analysis of the Canadian Cohort Obstructive Lung Disease (CanCOLD) study. We included healthy males and females aged ≥40 years who completed symptom limited incremental cycle CPET. The probability of each Borg CR10 leg discomfort rating by W or V'O_2_ was predicted using multinomial logistic regression. Model performance was evaluated by fit, calibration, discrimination (c‐statistic), and externally validated in an independent sample (*n* = 86) of healthy Canadian adults.

**Results:**

In total, 156 participants (43% female) were included (mean age 64.8 years). The models demonstrated good discrimination in both internal and external validation (AUC 0.85–0.90), with similar performance across absolute and relative W and V'O_2._ An upper limit of normal ([ULN]; 95th percentile) could not be defined, as leg discomfort responses were highly clustered within the predicted normal range across exercise intensities.

**Conclusions:**

We present normative reference equations for leg discomfort during CPET. Although an ULN could not be established, these models enable grading and interpretation of leg discomfort relative to the predicted normal responses and facilitate comparisons across individuals and groups in both clinical and research settings.

## 1 INTRODUCTION

Leg discomfort, assessed using the Borg category‐ratio 0–10 (Borg CR10) scale), is a primary symptom limiting exercise during incremental cardiopulmonary exercise testing (CPET) on a cycle ergometer in both healthy adults and individuals with cardiorespiratory disease, such as chronic obstructive pulmonary disease (Clark et al., 1995; Killian et al., 1992; Saey et al., 2003; Smyth et al., 2023; Tracey et al., 2020) or peripheral artery disease (Leeper et al., 2013). Previous work from our group has shown that participants self‐select leg discomfort (either alone or in conjunction with breathlessness) as the main reason for stopping exercise approximately ≥60% of the time (Muscat et al., 2015; Tracey et al., 2020). Despite this, there is currently no clear framework to determine whether a given level of leg discomfort at a specific exercise intensity is normal or abnormal. This represents an important knowledge gap, as interpretation of symptom‐limitation during CPET is central to understanding exercise intolerance (notably in those with chronic conditions), can guide clinical decision‐making, and be used to evaluate interventional effects. Establishing the normative leg discomfort response across increasing exercise intensities would enable clinicians and researchers to contextualize symptom intensity relative to healthy populations, rather than relying on absolute Borg CR10 ratings or absolute differences in ratings within or between subjects. Such reference values could be applied across cross‐sectional, observational, and interventional studies. Previous work by Killan and colleagues (Killian et al., 1992) attempted to create reference values for leg discomfort as a function of peak power output achieved during CPET; however, this analysis assumed normally distributed residuals and the use of linear regression, wherein predicted scores can fall outside of the 0–10 range, an important limitation previously outlined by Ekström et al. (2024). Centile‐based reference values for leg effort during incremental cycle ergometry have also been proposed, identifying the cumulative symptom response across exercise intensities to classify overall symptom burden (Hijleh et al., 2024). However, this approach does not provide a probabilistic framework to estimate the likelihood of a given leg effort or discomfort response at a specific exercise intensity.

Our group published normative reference equations for breathlessness intensity during incremental cycle CPET for healthy adults aged ≥18 and ≥40 years, respectively (Ekström et al., 2024; Elmberg et al., 2023). The latter was also validated in people with chronic airflow limitation (Ekström et al., 2024), and uniquely was also developed in relation to power output (W), rate of oxygen uptake (V'O_2_), and minute ventilation (V'_E_), allowing for an appreciation of the underlying pathophysiological mechanisms that may be contributing to exertional breathlessness (Stickland et al., 2022). As described by Stickland et al. (2022), abnormally high breathlessness for any given submaximal W and/or V'O_2_, but within normal limits for any given V'_E_, most likely points to an abnormally high ventilatory demand driving the breathlessness response. In contrast, abnormally high breathlessness for any given W, V'_E_, and/or V'O_2_ likely means there are dynamic respiratory mechanical factors, either alone or in conjunction with abnormally high ventilatory demand, driving the breathlessness response (Stickland et al., 2022). Thus, it is important to create normative reference equations for leg discomfort using the same concepts, albeit *sans* the respiratory component of V'_E_. Most importantly, the creation of normative reference values for both breathlessness and leg discomfort allows for the identification of the *presence* and the *level* of an abnormal response during standardized exercise intensities. As leg discomfort represents a key limiting factor in exercise tolerance alongside breathlessness, the development of similar reference norms is important: representing another “piece of the puzzle” in better understanding normal and abnormal perceptual symptom response during CPET.

The aims of this study were to: (i) develop normative reference equations for leg discomfort in healthy females and males aged ≥40 years during symptom‐limited incremental cycle CPET, in relation to absolute and relative (%predicted peak) W and V'O_2_, and (ii) validate these normative reference equations for leg discomfort in people with chronic airflow limitation.

## 2 MATERIALS AND METHODS

### 2.1 Study design and development sample

This was an analysis of the Canadian Cohort Obstructive Lung Disease (CanCOLD) study (Bourbeau et al., 2014). CanCOLD is a prospective, population‐based study conducted across nine communities in Canada (ClinicalTrials.gov Identifier: NCT00920348). Participants were noninstitutionalized male or female adults aged ≥40 years identified from the general population with random telephone digit dialling (Bourbeau et al., 2014).

Inclusion criteria for the development of the leg discomfort reference equations were healthy people aged ≥40 years with available CPET data from the CanCOLD baseline visit. A detailed list of exclusion criteria is found in the Supplemental Material; however, in brief, exclusion criteria included but was not limited to any known respiratory, cardiovascular, or metabolic disease (self‐reporting of physician‐diagnosed asthma, chronic bronchitis, chronic obstructive pulmonary disease, angina pectoris, myocardial infection, any other cardiovascular or cerebrovascular disease, or diabetes mellitus.

All participants provided written informed consent prior to completing study assessments. The research ethics board for each participating institution approved the study protocol. The current CanCOLD sub‐study is reported in accordance with the Transparent reporting of a multivariable prediction model for individual prognosis or diagnosis (TRIPOD) and the STrengthening the Reporting of OBservational studies in Epidemiology (STROBE) statements (Elm et al., 2007).

### 2.2 Assessment and procedures

All data were from CanCOLD visit 1. A detailed summary of all assessments and procedures has been published (Ekström et al., 2024; Lewthwaite et al., 2020). Briefly, participants self‐reported data on socio‐demographics and health, completed questionnaires assessing health‐related quality of life and physical activity habits, and performed pre‐ and post‐bronchodilator pulmonary function testing.

### 2.3 Cardiopulmonary exercise testing (CPET)

A detailed summary of all procedures related to CPET is described elsewhere (Ekström et al., 2024; Lewthwaite et al., 2020). In brief, CPET was performed on an electronically braked cycle ergometer using a computerized CPET system (Vmax, SensorMedics [seven sites], *n* = 138/156 participants [88.5%]; TrueOne, Parvomedics [one site] and Ergocard, Medisoft [one site], *n* = 18/156 participants [11.5%]) (American Thoracic S, and American College of Chest P., 2003). Participants cycled at an increasing power output of 10 W/min in a stepwise manner until symptom limitation (Lewthwaite et al., 2020). Gas exchange and breathing pattern parameters were collected breath‐by‐breath, while heart rate and rhythm and peripheral oxyhemoglobin saturation (SpO_2_) were continuously monitored. At rest, every 2 min during exercise, and at peak exercise, participants rated the intensity (magnitude) of their perceived breathlessness and leg discomfort using the modified Borg CR10 scale (Burdon et al., 1982). Prior to CPET, breathlessness was defined for each participant as ‘breathing discomfort’ and leg discomfort as ‘the level of discomfort experienced during pedalling’ and participants were familiarized with Borg's CR10 scale such that ‘0’ represented ‘no breathing/leg discomfort’ and ‘10’ represented ‘the most severe breathing/leg discomfort that you have ever experienced or can imagine experiencing.’

### 2.4 External validation sample

External validation was performed on an independent sample of 86 (49% female) ostensibly healthy participants aged ≥40 years, who performed an incremental cycle CPET to symptom limitation as part of studies independent from CanCOLD at the institutions of MKS (*n* = 27 from previous studies (Ross et al., 2020); Phillips et al., 2021) and DJ (*n* = 59; not included in previous studies). Exclusion criteria were abnormal lung function at rest (post‐bronchodilator forced expiratory volume in 1 s/forced vital capacity (FEV_1_/FVC) or FEV_1_ <lower limit of normal (<LLN)), body mass index (BMI) < 18 or >35 kg/m^2^, peak V'O_2_ < LLN (Lewthwaite et al., 2020), or missing data on peak leg discomfort. Symptom‐limited incremental CPETs were performed on an electronically braked cycle ergometer using a Vmax SensorMedics metabolic cart and included increments in power output of 15 W/2 min (*n* = 1), 20 W/2 min (*n* = 50), 20 W/3 min (*n* = 32), 25 W/2 min (*n* = 3), depending on the original study designs. Standardized physiological and symptom assessments were performed similarly to those in the CanCOLD development sample.

### 2.5 Analysis of leg discomfort responses for abnormality

An additional analysis in people aged ≥40 years with chronic airflow limitation was conducted to assess for normality of leg discomfort in relation to peak W and V'O_2_. The *probability of normality of the leg discomfort response* was defined as the predicted probability of each participant's Borg CR10 leg discomfort rating at peak exercise. This probability reflects how likely each participant's leg discomfort rating would be at any given peak W and V'O_2_, relative to a healthy reference population with similar age, sex, and body mass. A lower probability indicates that the observed response is less likely to occur in healthy individuals, and therefore more likely to reflect an abnormal leg discomfort response.

### 2.6 Statistical analyses

Baseline participant characteristics were summarized using mean with standard deviation (SD) and median with range or interquartile range (IQR) for continuous variables, as appropriate. Categorical variables were expressed as frequencies and percentages. No data were imputed. Leg discomfort ratings (Borg CR10) were analyzed uniquely for females and males, and by the two CPET parameters (W and V'O_2_), each evaluated as absolute values or as a percentage of each participant's predicted maximal value (%pred_max_) in separate models (Lewthwaite et al., 2020).

Normative reference equations were developed using CanCOLD data and marginal ordinal multinomial logistic regression. The models were fitted by a generalized estimating equation procedure with a cumulative logits link and multinomial distribution, to obtain population‐average (marginal) predictions. This method predicts the cumulative probability of reporting an equal or lower score for each of the Borg CR10 scores (0, 0.5, 1, 2…10). The ULN was calculated using linear interpolation of the linear predictor of the responses closest to below and above a probability of 0.95. The prediction equation was based on the CPET parameter and covariates and accounted for the correlation between repeated measurements on the same participant over the exercise time. In this way, no predictions fall outside the Borg CR10 scale range. We used LOESS (locally estimated scatterplot smoothing) plots to check the patterns between the Borg CR10 leg discomfort ratings and each of the CPET parameters. If the trend indicated non‐linearity, restricted cubic splines (Harrell, 2015) were applied with four knots, selected based on the distribution of the variables located at the 5th, 35th, 65th, and 95th percentile, constructed using the SAS macro %RCSPLINE (Harrell, 2004). Details on how to construct splines are given in the supplement.

The models were specified, and variables to include were selected using the independence model criterion (QIC), including comparing models with linear variables and cubic splines with four knots. Models with the lowest QIC were preferred. Results indicated that the models with four knots had a better fit for most of the variables (Supplemental Table S1). Additional factors (age, height, body mass, and their interaction terms with the two CPET parameters [W and V'O_2_]) with a *p* < 0.05 were also included in the final multivariate reference equations. For use in future validation studies, the distribution of each included variable according to the four knot cut points is shown in Supplemental Table S2.

Model performance in the development and validation samples was evaluated as calibration (agreement between predicted and observed probabilities for the different breathlessness scores) and discrimination. Calibration plots were created using the predicted probability by deciles on the *x*‐axis, and the observed rates by deciles on the y‐axis. A good calibration should lie close to the diagonal line of identify. The models were also validated by calculating the average absolute difference (observed‐predicted, %) between the predicted probabilities and observed frequencies. The discriminative ability of the model was assessed as the area under the curve (c‐statistic) of Receiver Operating Characteristic (ROC) analysis, indicating the probability of correct prediction of the different leg discomfort ratings. Statistical significance was defined as two‐sided *p*‐value < 0.05. Statistical analyses were conducted using the SAS version 9.4 software (TS1M5) (SAS Institute Inc., Cary, NC, USA, 2016).

## 3 RESULTS

### 3.1 Leg discomfort response

#### 3.1.1 Development of reference equations

**FIGURE 1:**
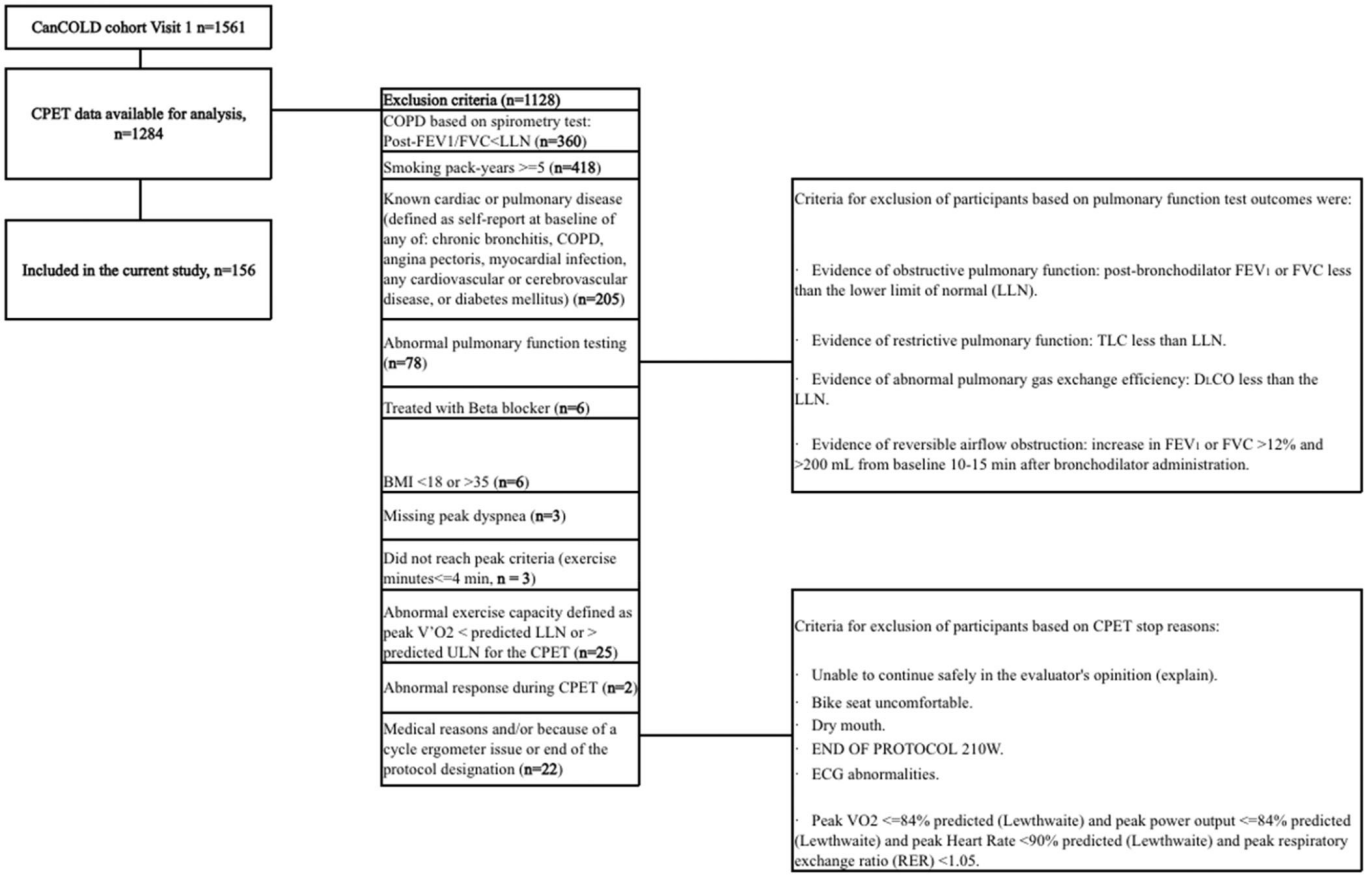
Participant flow chart in the CanCOLD development sample. BD, bronchodilation; BMI, body mass index; COPD, chronic obstructive pulmonary disease; CPET, cardiopulmonary exercise test; ECG, electrocardiogram; LLN, lower limit of normal; ULN, upper limit of normal; for other see abbreviations for Table 1.

Data from 156 CanCOLD participants (43% women) were used to develop the normative reference equations (Figure 1). Participant characteristics are shown in Table 1. The mean age was 64.8 years (range 42–91), BMI was 26.3 kg/m^2^ (SD 3.8), and lung function and peak physiological responses during CPET were within normal ranges (Table 1). CPET responses of all participants are shown in Table 1. Leg discomfort ratings at peak exercise were similar between males (median 6; IQR 5–9) and females (median 6; IQR 4–9). A leg discomfort rating of 10 was also achieved in a similar percentage of males (13.5%) and females (14.9%). In the entire cohort, 50% of participants cited leg discomfort as their main reason for stopping exercise, while 17.1% reported the combination of leg discomfort and breathlessness as their main reason for stopping exercise (Table 1).

**TABLE 1:** Participant characteristics and cardiopulmonary exercise test (CPET) responses of healthy adults in the development (CanCOLD) sample.

Characteristics	All	Male	Female
Participants, *n* (%)	156 (100)	89 (57)	67 (43)
Age, years, mean (SD) [min, max]	64.8 (9.5)	65.8 (9.5)	63.6 (9.3)
	[42.0, 91.0]	[47.0, 91.0]	[42.0, 81.0]
Height, cm	168.3 (9.5)	173.8 (7.4)	161.0 (6.5)
Body mass, kg	74.7 (14.1)	81.8 (12.3)	65.2 (10.3)
Body mass index, kg/m^2^	26.3 (3.8)	27.1 (3.8)	25.1 (3.6)
Cigarette ever smoker, *n* (%)	26 (16.7)	13 (14.6)	13 (19.4)
Cigarette smoker pack years	0.4 (1.1)	0.3 (1.1)	0.4 (1.1)
Hypertension, *n* (%)	33 (21.2)	20 (22.5)	13 (19.4)
CHAMPS moderate and greater intensity, Caloric expenditure per week (KC)	2864.4 (2604.7)	3407.6 (2938.7)	2142.9 (1868.6)
CHAMPS all activities, Caloric expenditure per week (KC)	4488.1 (3280.0)	5175.8 (3525.0)	3574.5 (2685.6)
Lung function			
FEV_1_, %pred	102.9 (13.4)	101.4 (12.0)	104.9 (14.9)
FVC, %pred	106.6 (14.2)	106.0 (13.5)	107.3 (15.1)
FEV_1_/FVC	75.1 (6.7)	73.8 (7.2)	76.9 (5.6)
TLC, %pred	105.5 (13.1)	102.0 (11.5)	110.1 (13.6)
RV, %pred	111.0 (26.8)	104.5 (26.6)	119.7 (24.7)
RV/TLC, %pred	104.5 (18.4)	101.9 (19.9)	107.9 (15.7)
D_L_CO, %pred	102.7 (16.6)	104.5 (17.3)	100.3 (15.5)
CPET values at peak exercise			
Work rate, W	131.0 (40.8)	150.4 (37.1)	105.2 (29.7)
W, %pred	102.2 (19.2)	101.8 (17.6)	102.7 (21.2)
HR, bpm	148 (20.4)	146 (21.8)	150 (18.4)
HR, %pred	100.6 (12.1)	99.9 (13.2)	101.6 (10.3)
V'O_2_, L/min	1.9 (0.6)	2.2 (0.5)	1.5 (0.4)
V'O_2_, %pred	100.3 (18.5)	98.1 (16.0)	103.2 (21.2)
V'O_2_, mL/kg/min	25.4 (6.2)	27.2 (5.7)	22.9 (6.0)
V'_E_, L/min	66.9 (19.8)	77.0 (18.2)	53.4 (12.4)
V'_E_, %pred	99.1 (23.2)	102.7 (23.8)	94.2 (21.6)
SBP, mm Hg	185.9 (27.1)	193.3 (24.4)	176.4 (27.7)
DBP, mm Hg	81 (12.1)	82 (12.2)	81 (12.1)
SpO_2_, %	96.8 (3.1)	96.3 (2.8)	97.4 (3.2)
RER	1.1 (0.1)	1.1 (0.1)	1.2 (0.1)
Breathlessness (Borg CR10), median (Q1, Q3)	5.0 (3.5, 7.0)	5.0 (3.0, 7.0)	5.0 (4.0, 7.0)
0, *n* (%)	3 (1.9)	1 (1.1)	2 (3.0)
0.5, *n* (%)	4 (2.6)	0 (0.0)	4 (6.0)
1, *n* (%)	4 (2.6)	4 (4.5)	0 (0.0)
2, *n* (%)	9 (5.8)	6 (6.7)	3 (4.5)
3, *n* (%)	19 (12.2)	12 (13.5)	7 (10.4)
4, *n* (%)	22 (14.1)	12 (13.5)	10 (14.9)
5, *n* (%)	31 (19.9)	18 (20.2)	13 (19.4)
6, *n* (%)	8 (5.1)	3 (3.4)	5 (7.5)
7, *n* (%)	22 (14.1)	11 (12.4)	11 (16.4)
8, *n* (%)	5 (3.2)	5 (5.6)	0 (0.0)
9, *n* (%)	23 (14.7)	12 (13.5)	11 (16.4)
10, *n* (%)	6 (3.8)	5 (5.6)	1 (1.5)
Leg discomfort (Borg CR10), median (Q1, Q3)	6.0 (4.0, 9.0)	6.0 (5.0, 9.0)	6.0 (4.0, 9.0)
0, *n* (%)	1 (0.6)	0 (0.0)	1 (1.5)
0.5, *n* (%)	1 (0.6)	1 (1.1)	0 (0.0)
1, *n* (%)	5 (3.2)	4 (4.5)	1 (1.5)
2, *n* (%)	4 (2.6)	1 (1.1)	3 (4.5)
3, *n* (%)	14 (9.0)	7 (7.9)	7 (10.4)
4, *n* (%)	18 (11.5)	6 (6.7)	12 (17.9)
5, *n* (%)	28 (17.9)	21 (23.6)	7 (10.4)
6, *n* (%)	8 (5.1)	5 (5.6)	3 (4.5)
7, *n* (%)	25 (16.0)	14 (15.7)	11 (16.4)
8, *n* (%)	6 (3.8)	3 (3.4)	3 (4.5)
9, *n* (%)	24 (15.4)	15 (16.9)	9 (13.4)
10, *n* (%)	22 (14.1)	12 (13.5)	10 (14.9)
Reasons for stopping CPET			
Leg discomfort	76 (50.0)	36 (41.9)	40 (60.6)
Breathlessness	14 (9.2)	7 (8.1)	7 (10.6)
Leg discomfort + breathlessness	26 (17.1)	19 (22.1)	7 (10.6)
Other	36 (23.7)	24 (27.9)	12 (18.2)

**FIGURE 2:**
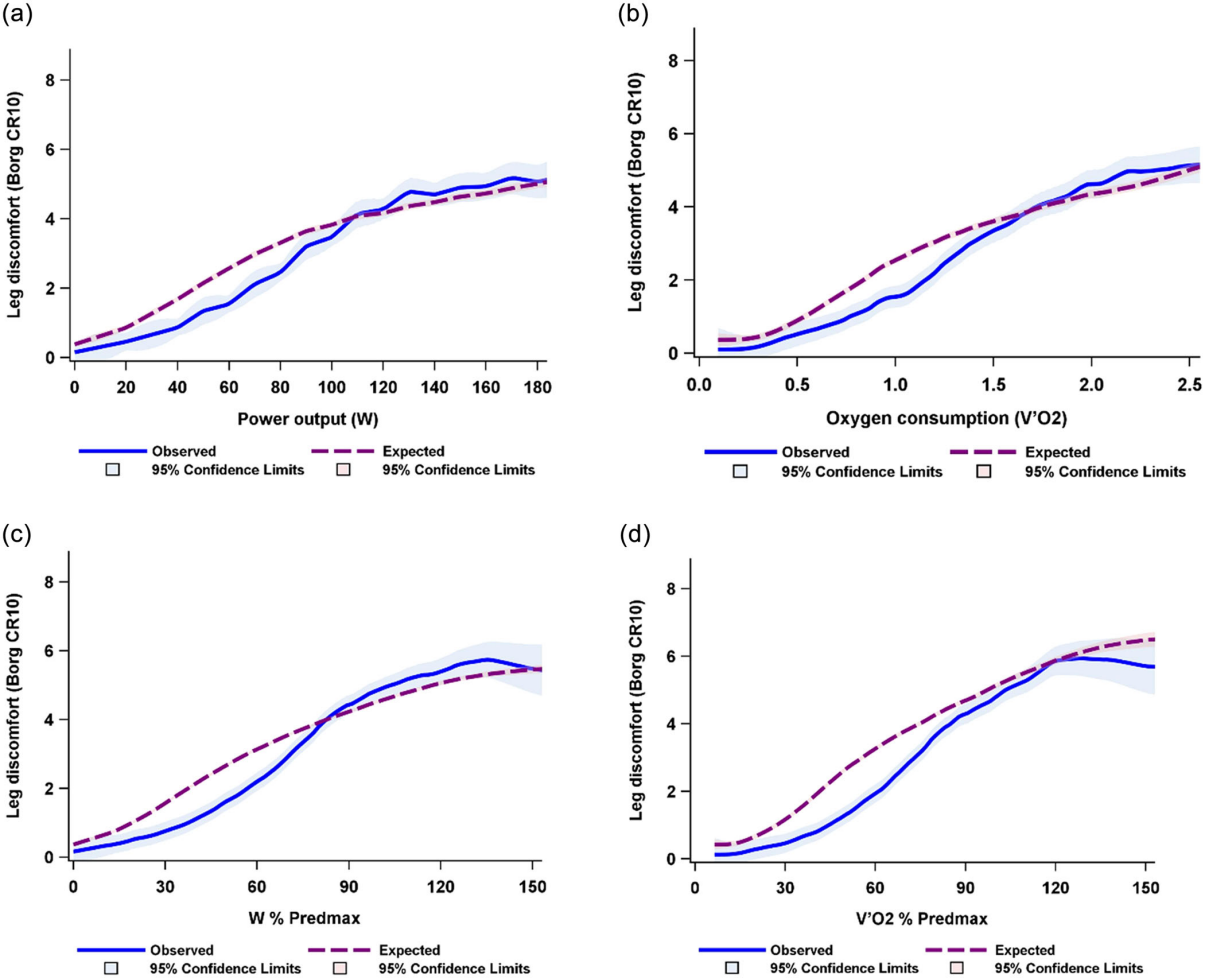
Observed and expected leg discomfort response during incremental cycle cardiopulmonary exercise testing in healthy males and females, plotted using penalized B‐spline by: (a) power output (W) expressed in absolute values; (b) rate of oxygen uptake (V'O_2_) expressed in absolute values; (c) W expressed in relative as a percentage of predicted maximum (%pred_max_) and; (d) V'O_2_ uptake relative as a percentage of %pred_max_. The expected leg discomfort is an anticipated average leg discomfort, calculated as the sum of all possible Borg CR10 scores, each multiplied by its predicted probability.

A penalized B‐spline was used to fit a smooth curve for the observed and expected leg discomfort ratings in the entire cohort (males and females) by absolute and relative W and V'O_2_ (Figure 2). The model suggests that at lower W and V'O_2_ intensities, the reference equations may slightly overestimate leg discomfort but were a good fit at higher intensities. The ULN (defined as the 95th percentile or scores, corresponding to a probability of normality < 0.05), could not be estimated as 99% of the cohort's leg discomfort responses were found to be <ULN, likely reflecting a clustering of responses within the upper bounds of the Borg CR10 scale (i.e., a ceiling effect), limiting discrimination between high but physiologically appropriate and truly abnormal leg discomfort responses. In the multivariate modelling, factors that improved the prediction of leg discomfort were age, sex, body weight, and height. The estimates for each factor are shown in Supplemental Table S3, and the goodness of fit for each model (assessed using the QIC) is shown in Supplemental Table S1.

#### 3.1.2 Internal validation

The prediction equations show good performance in terms of agreement (calibration) between predicted and observed probability (Supplemental Table S4 and Figure 2); however, at lower CPET values, the models slightly overestimate the Borg scores of leg discomfort (as mentioned above). Nevertheless, the models showed moderately strong discriminative ability (ROC curves shown in Supplemental Figure S1) in the entire cohort (AUC 0.85–‐0.90). The models performed similarly well when using the W and V'O_2,_ in both absolute values and %pred_max._

#### 3.1.3 External validation

**FIGURE 3:**
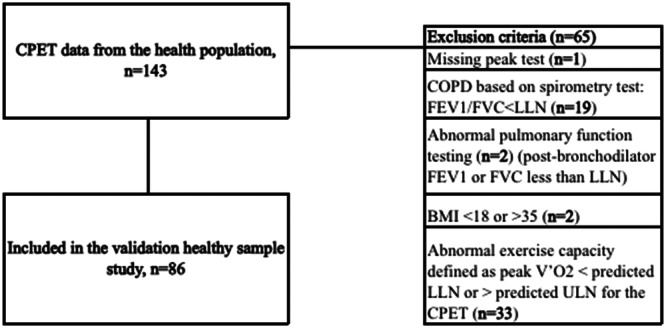
Participant flow chart in the external validation sample. For abbreviations, see Table 1.

The normative reference equations were applied to the external validation sample of healthy adults (*n* = 86) (Figure 3). This cohort had a mean age of 68.3 years (SD 9.9), a mean BMI of 26.0 km/m^2^ (SD 3.3), with lung function and exercise capacity within normal ranges. 49% of the external validation cohort were female (Table 2). Leg discomfort ratings at peak exercise were higher in males (median 7; IQR 4–8) than in females (median 5; IQR 4–7) (Table 3).

**TABLE 2:** Participant characteristics of the external validation sample of healthy adults.

Characteristic	All	Male	Female
Participants, *n* (%)	86 (100)	44 (51)	42 (49)
Age, mean (SD), range	68.3 (9.9) [41.0, 91.0]	68.7 (11.0) [41.0, 91.0]	67.8 (8.7) [42.0, 80.0]
Height, cm	167.6 (9.4)	173.6 (7.4)	161.4 (6.9)
Body mass, kg	73.4 (13.1)	81.2 (11.3)	65.2 (9.4)
Body mass index, kg/m^2^	26.0 (3.3)	27.0 (3.2)	25.0 (3.2)
Current smoker, *n* (%)	15 (17.4)	4 (9.1)	11 (26.2)
Lung function			
FEV_1_, %pred	102.9 (12.9)	102.0 (13.8)	103.7 (11.9)
FVC, %pred	106.5 (14.4)	105.5 (15.8)	107.5 (13.0)
FEV_1_/FVC, %	0.7 (0.1)	0.7 (0.1)	0.8 (0.1)

**TABLE 3:** Cardiopulmonary exercise test (CPET) responses of external validation cohort of healthy adults.

CPET values at peak exercise	All	Male	Female
Power output, W	133.7 (44.9)	152.7 (44.2)	113.8 (36.5)
W, %pred	114.1 (26.5)	107.8 (22.6)	120.7 (28.9)
V'O_2_, L/min	2.0 (0.6)	2.3 (0.5)	1.6 (0.4)
V'O_2_, %pred	112.9 (20.2)	106.4 (17.5)	119.6 (20.8)
V'_E_, L/min	74.9 (22.9)	84.7 (23.5)	64.7 (17.3)
V'_E_, %pred	117.0 (27.6)	115.3 (28.6)	118.7 (26.8)
Breathlessness (Borg CR10), median (Q1, Q3)	4.0 (3.0, 7.0)	6.0 (3.0, 7.0)	4.0 (3.0, 7.0)
0, *n* (%)	1 (1.2)	0 (0.0)	1 (2.4)
0.5, *n* (%)	2 (2.3)	1 (2.3)	1 (2.4)
1, *n* (%)	3 (3.5)	1 (2.3)	2 (4.8)
2, *n* (%)	3 (3.5)	2 (4.5)	1 (2.4)
3, *n* (%)	18 (20.9)	9 (20.5)	9 (21.4)
4, *n* (%)	15 (17.4)	5 (11.4)	10 (23.8)
5, *n* (%)	6 (7.0)	2 (4.5)	4 (9.5)
6, *n* (%)	3 (3.5)	3 (6.8)	0 (0.0)
7, *n* (%)	15 (17.4)	10 (22.7)	5 (11.9)
8, *n* (%)	5 (5.8)	4 (9.1)	1 (2.4)
9, *n* (%)	9 (10.5)	5 (11.4)	4 (9.5)
10, *n* (%)	3 (3.5)	0 (0.0)	3 (7.1)
Leg discomfort (Borg CR10), median (Q1, Q3)	6.0 (4.0, 7.5)	7.0 (4.0, 8.0)	5.0 (4.0, 7.0)
0, *n* (%)	0 (0.0)	0 (0.0)	0 (0.0)
0.5, *n* (%)	1 (1.2)	0 (0.0)	1 (2.4)
1, *n* (%)	2 (2.3)	0 (0.0)	2 (4.8)
2, *n* (%)	1 (1.2)	1 (2.3)	0 (0.0)
3, *n* (%)	12 (14.0)	7 (15.9)	5 (11.9)
4, *n* (%)	12 (14.0)	4 (9.1)	8 (19.0)
5, *n* (%)	13 (15.1)	4 (9.1)	9 (21.4)
6, *n* (%)	5 (5.8)	2 (4.5)	3 (7.1)
7, *n* (%)	17 (19.8)	13 (29.5)	4 (9.5)
8, *n* (%)	6 (7.0)	4 (9.1)	2 (4.8)
9, *n* (%)	5 (5.8)	2 (4.5)	3 (7.1)
10, *n* (%)	10 (11.6)	6 (13.6)	4 (9.5)

**FIGURE 4:**
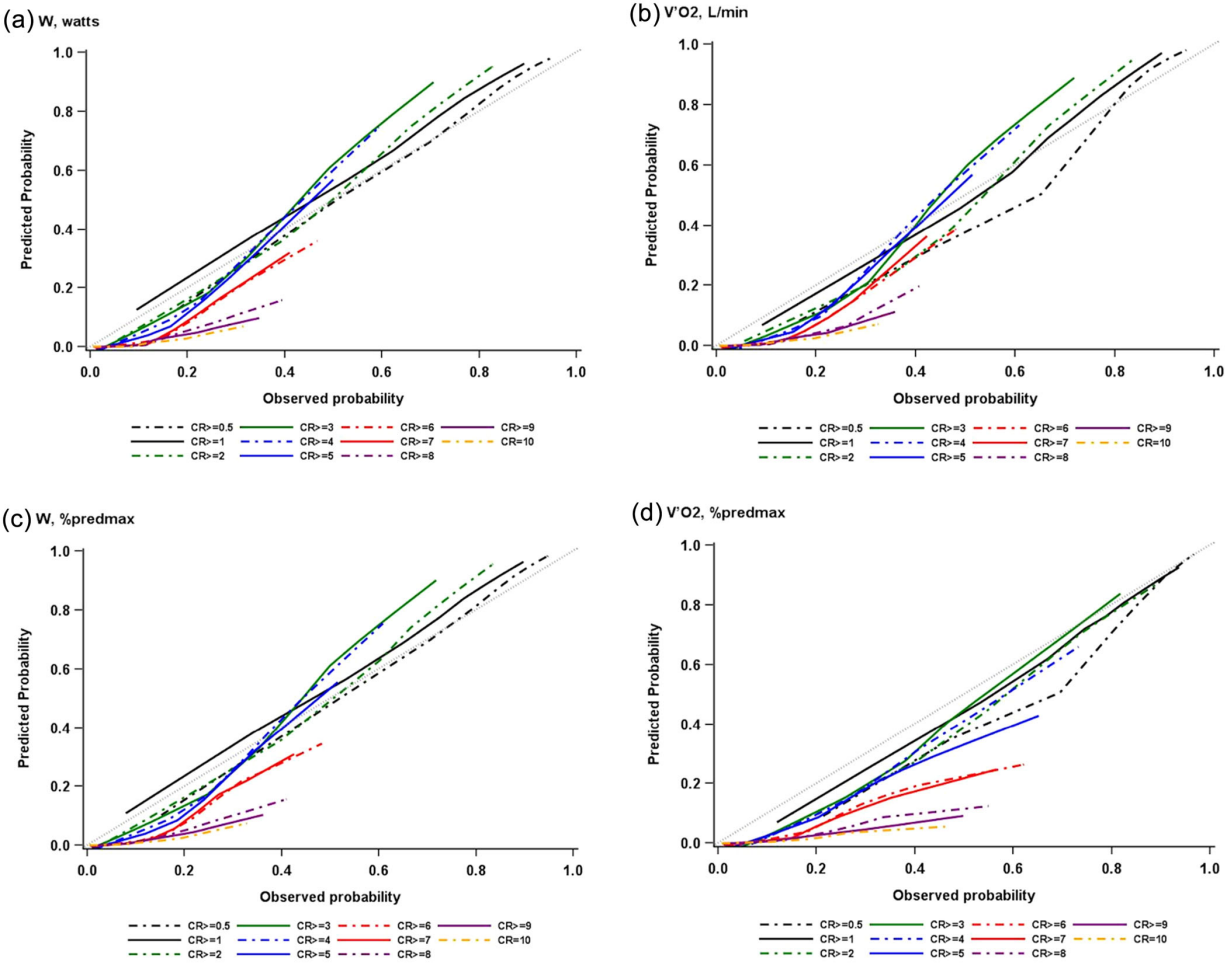
Calibration plots of the model performance in the external validation sample of healthy adults. Calibration demonstrates the agreement between observed outcomes and predictions (compared the empirical probability of the response to the predicted probability), and it can help diagnose lack of fit. The calibration plots were created using the predicted probability by deciles on the *x*‐axis, and the observed rates by deciles on the y‐axis. A good calibration should lie close to the 45‐degree line of identity; that is, the smoother lies close to the diagonal. If there is a systematic deviation from the diagonal line, that indicates that the model might be misspecified. Models were evaluated based on (a) absolute power output (W); (b) relative (%pred_max_) power output (W), (c) absolute rate of oxygen uptake (V'O_2_), and (d) relative V'O_2_ during symptom‐limited, cycle, incremental cardiopulmonary exercise testing (CPET).

Performance of the normative reference equations in the external validation sample was determined as moderately good and similar to that observed in the CanCOLD development sample for all equations (Table 4). The normal reference values were moderately well calibrated (Figure 4), with moderately discriminative ability to predict the leg discomfort ratings (AUC 0.78–0.88) (Supplemental Figure S2), albeit a lower discriminative ability than in the internal validation cohort.

**TABLE 4:** Fit of the normative reference equations in the external validation sample of healthy adults, in terms of average absolute difference (observed‐predicted, %) in probabilities for each leg discomfort (Borg CR10) score in males and females together.

Borg CR10 score	Normative reference equation			
	W, watts	V'O_2_, L/min	W, %predmax	V'O_2_, %predmax
^3^0.5	1.11 ± 5.84	−1.85 ± 8.46	0.73 ± 5.52	−6.05 ± 7.29
^3^1	5.50 ± 4.80	2.00 ± 5.64	4.98 ± 5.08	−3.38 ± 5.53
^3^2	3.24 ± 8.71	−0.27 ± 9.97	2.63 ± 9.38	−6.55 ± 5.24
^3^3	4.62 ± 11.53	1.69 ± 12.95	3.94 ± 11.77	−5.33 ± 6.33
^3^4	0.11 ± 8.79	−2.14 ± 9.82	−0.56 ± 8.67	−9.10 ± 3.85
^3^5	−3.87 ± 5.75	−5.60 ± 6.05	−4.48 ± 5.57	−12.14 ± 6.10
^3^6	−8.64 ± 4.77	−10.20 ± 4.69	−9.22 ± 5.58	−16.52 ± 10.23
^3^7	−8.10 ± 4.56	−9.39 ± 4.92	−8.62 ± 5.64	−15.25 ± 9.57
^3^8	−11.48 ± 7.96	−12.69 ± 7.40	−11.97 ± 8.42	−18.41 ± 12.87
^3^9	−10.89 ± 8.30	−11.90 ± 7.90	−11.34 ± 8.55	−17.15 ± 12.59
10	−10.48 ± 8.30	−11.37 ± 8.06	−10.89 ± 8.55	−16.26 ± 12.53

### 3.2 Normal leg discomfort response

#### 3.2.1 Analysis of the probability of leg discomfort normality in people with chronic airflow limitation

The probability of leg discomfort normality was assessed in an external cohort of people with chronic airflow limitation (*n* = 330). Fewer than 1% of individuals were classified as having an abnormal leg discomfort response (i.e., >ULN), indicating that most responses fell within the predicted normal range defined by the model. For a given level of W, only 1 participant was >ULN, while for a given level of V'O_2_, only five participants were >ULN. Any additional analyses in this cohort were not pursued.

## 4 DISCUSSION

We present normative reference equations for evaluating leg discomfort response during symptom‐limited incremental cycle CPET, developed and validated in healthy Canadian males and females aged ≥40 years. The primary findings are as follows. First, the models demonstrated good agreement between observed and predicted leg discomfort across exercise intensities in males and females, with slight overestimation at lower levels of W and V'O_2_ (∼30–60% W and V'O_2%_pred_max_). Second, despite good model performance, a ULN could not be defined, as leg discomfort ratings were highly clustered within the upper bounds of the Borg CR10 scale across all levels of W and V'O_2_. This indicates that high levels of leg discomfort are common and physiologically appropriate in response to incremental exercise on a cycle ergometer in healthy people. Consequently, leg discomfort during CPET may be better interpreted relative to expected values (i.e., using the normative reference equations as is) rather than using dichotomous normal/abnormal thresholds, in contrast to previously developed models for breathlessness intensity during CPET (Ekström et al., 2024). The observed clustering may also be influenced by measurement characteristics of the Borg CR10 scale at higher symptom intensities, where the absence of verbal descriptors at ratings 6 and 8 may reduce its ability to discriminate between high levels of perceived leg discomfort (Aucoin et al., 2025). Future studies should also explore whether alternative symptom scales (e.g., Numerical Rating Scale) with greater resolution at higher symptom intensities improve discrimination of leg discomfort at high or near‐max exercise intensities. Finally, although only <15% of individuals reported a maximal leg discomfort rating of 10 on the Borg CR10 scale, the majority of participants identified leg discomfort as the primary reason for exercise cessation. This is consistent with contemporary literature on symptom responses during CPET and further reinforces the central role of leg discomfort in exercise limitation. Taken together, these findings support the concept that leg discomfort is an expected and dominant perceptual response during CPET, highlighting the need for context‐specific interpretation rather than reliance on fixed dichotomous thresholds or normal versus abnormal.

Our findings indicate that while leg discomfort responses during CPET can be predicted across exercise intensities, defining a ULN may not be appropriate in this context. This does not diminish the utility of these reference equations; rather, they provide a framework to contextualize leg discomfort responses relative to normative values in healthy populations. This enables comparison across individuals and groups, both prospectively and retrospectively, and may not be particularly relevant in populations where exaggerated leg discomfort is anticipated due to underlying pathophysiology such as peripheral artery disease (Leeper et al., 2013). Despite a ULN not being established in the present study, future studies could evaluate clinically meaningful probability‐of‐normality thresholds linked to patient‐important outcomes (e.g., hospitalizations, mortality) to improve the clinical interpretation of leg discomfort during CPET. As for the external validation in people with chronic airflow limitation (n = 330), the vast majority of responses (>99%) fell within the predicted normal range. This suggests that high levels of leg discomfort are commonly observed even in this population and may reflect a typical response to incremental cycle exercise rather than clear evidence of abnormality. One could speculate that in healthy recreationally active or sedentary adults, cycle exercise inherently causes a high level of leg discomfort, and that it is normal for these individuals to terminate exercise solely due, or at least in‐part due to leg discomfort (Muscat et al., 2015; Tracey et al., 2020).

### 4.1 Strengths and limitations

Strengths of this study include the use of CanCOLD, which is a well‐characterized, multicenter population‐based sample. CanCOLD comprises male and female participants who have completed standardized symptom‐limited incremental CPETs with detailed monitoring of breath‐by‐breath cardiac, metabolic, and respiratory parameters and subjective symptom responses. Additional detailed assessments of lung function, in accordance with ATS/ERS standards (American Thoracic S, and American College of Chest P., 2003; Stanojevic et al., 2022), are also included in CanCOLD. We recognize that our relatively small sample size (*n* = 156), primarily limited by our detailed list of inclusion and exclusion criteria used to ensure a healthy reference population that achieved symptom‐limitation during CPET, may have increased the risk of model overfitting given the complexity of the statistical approach. However, model performance was assessed using both internal and external validation, with consistently good calibration and discrimination across cohorts. Although the external validation cohort completed CPETs using different stage durations and power output increments, all tests were performed using symptom‐limited incremental cycle ergometry, and model performance remained similar across cohorts. Furthermore, the equations were evaluated in an additional external cohort of 330 individuals with chronic airflow limitation, supporting the robustness and generalizability of the models despite the modest development sample.

### 4.2 Conclusions

These normative reference equations allow for prediction of leg discomfort responses during incremental cycle CPET in healthy older adults aged ≥40 years, as well as in adults with chronic airflow limitation (albeit with some limitations as described above). Although a ULN could not be defined, this finding reflects the high and expected levels of leg discomfort observed across exercise intensities, rather than a limitation of the models. Accordingly, these equations provide a framework for clinicians and researchers to compare an individual's observed leg discomfort response with the expected response for a healthy person of similar age, sex, and body mass at a given exercise intensity, supporting individualized interpretation of symptom responses and comparisons within and between individuals or groups. Given that leg discomfort is a primary determinant of exercise cessation across health and disease (Muscat et al., 2015; Saey et al., 2003), these reference equations (alongside existing normative reference equations for breathlessness) offer a more comprehensive approach to evaluating perceptual responses to exercise and may enhance the interpretation of symptom limitation in both clinical and research settings.

## CONFLICT OF INTEREST STATEMENT

Jean Bourbeau and Wan C. Tan report receiving institutional funding for the CanCOLD study from AstraZeneca Canada Ltd., Boehringer‐Ingelheim Canada Ltd, GlaxoSmithKline Canada Ltd, Merck, Novartis Pharma Canada Inc., as well as Nycomed Canada Inc. (Wan C. Tan), Pfizer Canada Ltd. (Wan C. Tan), Trudell (Jean Bourbeau), and Grifolds (Jean Bourbeau). No competing interests exist for any of the other authors.

## Supporting information

Supporting File 1.

## Data Availability

The data that support the findings of this study are available from the corresponding author upon reasonable request.

## References

[cpf70083-bib-0001] American Thoracic S, and American College of Chest P . (2003) ATS/ACCP Statement on cardiopulmonary exercise testing. American Journal of Respiratory and Critical Care Medicine, 167(2), 211–277. 12524257 10.1164/rccm.167.2.211

[cpf70083-bib-0002] Aucoin, R. , Ekström, M. , Li, P.Z. , Bourbeau, J. , Tan, W.C. & Jensen, D. (2025) Response bias for modified Borg 0‐10 numerical categories without verbal descriptors during assessment of exertional symptoms. American Journal of Respiratory and Critical Care Medicine, 211(2), 284–287. 39514843 10.1164/rccm.202406-1206RL

[cpf70083-bib-0003] Bourbeau, J. , Tan, W.C. , Benedetti, A. , Aaron, S.D. , Chapman, K.R. , Coxson, H.O. et al. (2014) Canadian cohort obstructive lung disease (CanCOLD): fulfilling the need for longitudinal observational studies in COPD. COPD: Journal of Chronic Obstructive Pulmonary Disease, 11(2), 125–132. 22433011 10.3109/15412555.2012.665520

[cpf70083-bib-0004] Burdon, J.G. , Juniper, E.F. , Killian, K.J. , Hargreave, F.E. & Campbell, E.J. (1982) The perception of breathlessness in asthma. The American Review of Respiratory Disease, 126(5), 825–828. 7149447 10.1164/arrd.1982.126.5.825

[cpf70083-bib-0005] Clark, A.L. , Sparrow, J.L. & Coats, A.J.S. (1995) Muscle fatigue and dyspnoea in chronic heart failure: two sides of the same coin? European Heart Journal, 16(1), 49–52. 10.1093/eurheartj/16.1.49 7737221

[cpf70083-bib-0006] Ekström, M. , Li, P.Z. , Lewthwaite, H. , Bourbeau, J. , Tan, W.C. , Jensen, D. et al. (2024) Abnormal exertional breathlessness on cardiopulmonary cycle exercise testing in relation to self‐reported and physiologic responses in chronic airflow limitation. Chest, 166(1), 81–94. 38423279 10.1016/j.chest.2024.02.034

[cpf70083-bib-0007] Ekström, M. , Li, P.Z. , Lewthwaite, H. , Bourbeau, J. , Tan, W.C. & Schiöler, L. et al. (2024) Normative reference equations for breathlessness intensity during incremental cardiopulmonary cycle exercise testing. Annals of the American Thoracic Society, 21, 56–67. 37708387 10.1513/AnnalsATS.202305-394OC PMC10867914

[cpf70083-bib-0008] Elm, E. , Altman, D.G. , Egger, M. , Pocock, S.J. , Gøtzsche, P.C. & Vandenbroucke, J.P. (2007) Strengthening the reporting of observational studies in epidemiology (STROBE) statement: guidelines for reporting observational studies. BMJ, 335(7624), 806–808. 17947786 10.1136/bmj.39335.541782.AD PMC2034723

[cpf70083-bib-0009] Elmberg, V. , Schiöler, L. , Lindow, T. , Hedman, K. , Malinovschi, A. , Lewthwaite, H. et al. (2023) Reference equations for breathlessness during incremental cycle exercise testing. ERJ Open Research, 9(2), 00566–2022. 37057086 10.1183/23120541.00566-2022 PMC10086693

[cpf70083-bib-0010] Harrell, F.E. (2004) SAS Macros for Assisting with Survival and Risk Analysis, and Some SAS Procedures Useful for Multivariable Modeling. Available at http://biostat.mc.vanderbilt.edu/wiki/Main/SasMacros

[cpf70083-bib-0011] Harrell, F.E. (2015) Regression modeling strategies: with applications to linear models, logistic and ordinal regression, and survival analysis. Switzerland AG: Springer Nature.

[cpf70083-bib-0012] Hijleh, A.A. , Wang, S. , Berton, D.C. , Neder‐Serafini, I. , Vincent, S. , James, M. et al. (2024) Reference values for leg effort during incremental cycle ergometry in non‐trained healthy men and women, aged 19–85. Scandinavian Journal of Medicine & Science in Sports, 34(4), e14625. 38597357 10.1111/sms.14625

[cpf70083-bib-0013] Killian, K.J. , Summers, E. , Jones, N.L. & Campbell, E.J.M. (1992) Dyspnea and leg effort during incremental cycle ergometry. American Review of Respiratory Disease, 145(6), 1339–1345. 1596000 10.1164/ajrccm/145.6.1339

[cpf70083-bib-0014] Leeper, N.J. , Myers, J. , Zhou, M. , Nead, K.T. , Syed, A. , Kojima, Y. et al. (2013) Exercise capacity is the strongest predictor of mortality in patients with peripheral arterial disease. Journal of Vascular Surgery, 57(3), 728–733. 23044259 10.1016/j.jvs.2012.07.051 PMC3543469

[cpf70083-bib-0015] Lewthwaite, H. , Benedetti, A. , Stickland, M.K. , Bourbeau, J. , Guenette, J.A. , Maltais, F. et al. (2020) Normative peak cardiopulmonary exercise test responses in Canadian adults aged ≥40 years. Chest, 158(6), 2532–2545. 32679236 10.1016/j.chest.2020.06.074

[cpf70083-bib-0016] Lewthwaite, H. , Benedetti, A. , Stickland, M.K. , Bourbeau, J. , Guenette, J.A. , Maltais, F. et al. (2020) Normative peak cardiopulmonary exercise test responses in Canadian adults aged >/=40 years. Chest, 158(6), 2532–2545. 32679236 10.1016/j.chest.2020.06.074

[cpf70083-bib-0017] Muscat, K.M. , Kotrach, H.G. , Wilkinson‐Maitland, C.A. , Schaeffer, M.R. , Mendonca, C.T. & Jensen, D. (2015) Physiological and perceptual responses to incremental exercise testing in healthy men: effect of exercise test modality. Applied Physiology, Nutrition and Metabolism, 40(11), 1199–1209. 10.1139/apnm-2015-0179 26501683

[cpf70083-bib-0018] Phillips, D.B. , Brotto, A.R. , Ross, B.A. , Bryan, T.L. , Wong, E.Y.L. , Meah, V.L. et al. (2021) Inhaled nitric oxide improves ventilatory efficiency and exercise capacity in patients with mild COPD: a randomized‐control cross‐over trial. The Journal of Physiology, 599(5), 1665–1683. 33428233 10.1113/JP280913

[cpf70083-bib-0019] Ross, B.A. , Brotto, A.R. , Fuhr, D.P. , Phillips, D.B. , van Diepen, S. , Bryan, T.L. et al. (2020) The supine position improves but does not normalize the blunted pulmonary capillary blood volume response to exercise in mild COPD. Journal of Applied Physiology, 128(4), 925–933. 32163328 10.1152/japplphysiol.00890.2019

[cpf70083-bib-0020] Saey, D. , Debigaré, R. , LeBlanc, P. , Mador, M.J. , Côté, C.H. , Jobin, J. et al. (2003) Contractile leg fatigue after cycle exercise. American Journal of Respiratory and Critical Care Medicine, 168(4), 425–430. 12714348 10.1164/rccm.200208-856OC

[cpf70083-bib-0021] Smyth, R.M. , James, M.D. , Vincent, S.G. , Milne, K.M. , Marillier, M. , Domnik, N.J. et al. (2023) Systemic determinants of exercise intolerance in patients with fibrotic interstitial lung disease and severely impaired D(LCO). Respiratory Care, 68(12), 1662–1674. 37643871 10.4187/respcare.11147 PMC10676244

[cpf70083-bib-0022] Stanojevic, S. , Kaminsky, D.A. , Miller, M.R. , Thompson, B. , Aliverti, A. , Barjaktarevic, I. et al. (2022) ERS/ATS technical standard on interpretive strategies for routine lung function tests. European Respiratory Journal, 60(1), 2101499. 34949706 10.1183/13993003.01499-2021

[cpf70083-bib-0023] Stickland, M.K. , Neder, J.A. , Guenette, J.A. , O'Donnell, D.E. & Jensen, D. (2022) Using cardiopulmonary exercise testing to understand dyspnea and exercise intolerance in respiratory disease. Chest, 161(6), 1505–1516. 35065052 10.1016/j.chest.2022.01.021

[cpf70083-bib-0024] Tracey, L. , Lewthwaite, H. , Abdallah, S.J. , Murray, S. , Wilkinson‐Maitland, C.A. , Donovan, A. et al. (2020) Physiological and perceptual responses to exercise according to locus of symptom limitation in COPD. Respiratory Physiology & Neurobiology, 273, 103322. 31629879 10.1016/j.resp.2019.103322

