# Supplementary material for: Normative reference equations for leg discomfort during incremental cardiopulmonary cycle exercise testing in older adults
Source: Clin Physiol Funct Imaging. 2026 Jul 23;46(4):e70083. doi: 10.1111/cpf.70083 (PMC13393339; doi:10.1111/cpf.70083)
Supplement: Supplementary file 1 — Supporting File 1. [file CPF-46-0-s001.docx]

Supplemental Material

**Normative reference equations for leg discomfort during incremental cardiopulmonary cycle exercise testing in older adults**

Rachelle Aucoin^1^, Dennis Jensen^2,3^, Michael Stickland^4^, Andrew Brotto^4^, Hayley Lewthwaite^5^, Pei Zhi Li^6^, Jean Bourbeau^3,6^, Wan C. Tan^7^, Magnus Ekström^8^

On behalf of the CanCOLD Collaborative Research Group *

1. Respiratory Epidemiology Team, Faculty of Medicine, Dalhousie University, Halifax, NS, Canada.
2. Clinical Exercise and Respiratory Physiology Laboratory, Department of Kinesiology & Physical Education, McGill University, Montréal, QC, Canada.
3. Research Institute of the McGill University Health Centre, Translational Research in Respiratory Diseases Program and Respiratory Epidemiology and Clinical Research Unit, Montréal, QC, Canada
4. Division of Pulmonary Medicine, Faculty of Medicine and Dentistry, University of Alberta, Edmonton, AB, Canada; G.F. MacDonald Centre for Lung Health, Covenant Health, Edmonton, AB, Canada.
5. UniSA: Allied Health and Human Performance, Innovation, Implementation and Clinical Translation in Health, University of South Australia, Adelaide, Australia.
6. Montreal Chest Institute, McGill University Health Center Research Institute, McGill University, Montréal, Québec, Canada
7. University of British Columbia Centre for Heart Lung Innovation, Department of Medicine, Vancouver, BC, Canada
8. Lund University, Faculty of Medicine, Department of Clinical Sciences Lund, Respiratory Medicine, Allergology and Palliative Medicine, Lund, Sweden

**Methods**

Exclusion criteria were: known respiratory, cardiovascular or metabolic disease (self-reporting of physician-diagnosed asthma, chronic bronchitis, COPD, angina pectoris, myocardial infection, any other cardiovascular or cerebrovascular disease, or diabetes mellitus); treatment with beta blocker; ≥5 pack-years of cigarette smoke exposure; abnormally low or high exercise capacity, defined as peak V’O_2_ below the lower limit of normal [<LLN] or >ULN, respectively (1); and increase in forced expired volume in 1 second (FEV_1_) or forced vital capacity (FVC) >12% and >200 mL from baseline 10-15 min after inhalation of 200 μg of salbutamol administered *via* a spacer; and impaired lung function at rest, defined as post-bronchodilator value <LLN of the following measurements: FEV_1_, FVC (2), total lung capacity (TLC) (3), or diffusing capacity of the lungs for carbon monoxide (D_L_CO) (4); body mass index (BMI) <18 or >35 kg/m^2^; inability to reach peak exercise criteria exercise time <4 minutes; abnormal response during CPET as judged by the supervising physician; missing peak leg discomfort; or termination of the CPET by the supervising physician due to medical or technical reasons or end of the protocol designation.

**Table S1.** Independent model criterion (QIC) from univariate GEE models stratified in both males and females in the development (CanCOLD) sample. The model with smaller statistic was preferred.

|  | **Restricted cubic splines with 4 knots** | **One linear variable** |
| --- | --- | --- |
| W, absolute | **4092.953** | 4144.6044 |
| W, %pred_max_ | **4010.7966** | 4017.4203 |
| VO_2_, absolute | **4210.3704** | 4266.3336 |
| VO_2_, %pred_max_ | **4104.6914** | 4116.8698 |
| Age, years | 4915.7896 | **4911.8433** |
| Height, cm | **4917.3879** | 4919.3244 |
| Body mass, kg | 4924.4092 | **4918.8434** |

*Abbreviations:* W = watt; VO_2_ = rate of oxygen uptake; %pred_max_ = percentage of predicted maximum; cm = centimeter; kg = kilogram.

**Table S2.** Distribution of each variable according to the four knot cut points (percentiles) used in the final normative reference equations.

| **Variables** | **P5** | **P35** | **P65** | **P95** |
| --- | --- | --- | --- | --- |
| W, absolute | 0 | 40 | 100 | 160 |
| W, %pred_max_ | 0 | 35.1335 | 70.7632 | 113.42 |
| VO_2_, absolute | 0.26 | 0.843 | 1.4 | 2.38 |
| VO_2_, %pred_max_ | 14.2618 | 44.9567 | 72.3526 | 111.483 |
| Age, years | 47 | 60 | 67 | 79 |
| Height, cm | 154 | 166 | 174 | 185 |
| Body mass, kg | 56 | 68 | 83 | 98 |

*Abbreviations:* W = watt; VO_2_ = rate of oxygen uptake; %pred_max_ = percentage of predicted maximum; cm = centimeter; kg = kilogram.

**Table S3.** Parameter estimates for each normative reference equation.

| **Parameter** | **Estimate** | **SE** | **P-value** |
| --- | --- | --- | --- |
| **W, absolute** |  |  |  |
| Intercept1 | 5.589 | 4.0893 | 0.172 |
| Intercept2 | 6.3155 | 4.096 | 0.123 |
| Intercept3 | 6.8671 | 4.0951 | 0.094 |
| Intercept4 | 7.5603 | 4.1015 | 0.065 |
| Intercept5 | 8.0578 | 4.1025 | 0.050 |
| Intercept6 | 8.4378 | 4.1087 | 0.040 |
| Intercept7 | 8.5684 | 4.1104 | 0.037 |
| Intercept8 | 8.7999 | 4.1102 | 0.032 |
| Intercept9 | 8.864 | 4.1097 | 0.031 |
| Intercept10 | 9.0667 | 4.1113 | 0.027 |
| Intercept11 | 9.2129 | 4.1068 | 0.025 |
| W, absolute | -0.0583 | 0.0065 | <0.001 |
| W, absolute_Spl2 | 0.0846 | 0.0238 | <0.001 |
| W, absolute_Spl3 | -0.1315 | 0.0482 | 0.006 |
| Age | -0.0165 | 0.0084 | 0.050 |
| Sex (male=1, female=0) | 0.1629 | 0.1937 | 0.301 |
| Height | -0.0215 | 0.025 | 0.391 |
| Height_Spl2 | 0.1575 | 0.0698 | 0.024 |
| Height_Spl3 | -0.6448 | 0.2697 | 0.017 |
| **V'O2, absolute** |  |  |  |
| Intercept1 | 3.5131 | 4.5727 | 0.442 |
| Intercept2 | 4.1943 | 4.5794 | 0.360 |
| Intercept3 | 4.7202 | 4.5769 | 0.302 |
| Intercept4 | 5.3987 | 4.5779 | 0.238 |
| Intercept5 | 5.897 | 4.5797 | 0.198 |
| Intercept6 | 6.2807 | 4.5864 | 0.171 |
| Intercept7 | 6.4126 | 4.5878 | 0.162 |
| Intercept8 | 6.647 | 4.5893 | 0.148 |
| Intercept9 | 6.712 | 4.5891 | 0.144 |
| Intercept10 | 6.9171 | 4.589 | 0.132 |
| Intercept11 | 7.0645 | 4.5867 | 0.124 |
| V'O2, absolute | -4.8619 | 0.5031 | <0.001 |
| V'O2, absolute_Spl2 | 8.3339 | 1.7911 | <0.001 |
| V'O2, absolute_Spl3 | -16.9447 | 4.4577 | <0.001 |
| Sex (male=1, female=0) | 0.2392 | 0.1875 | 0.102 |
| Height | -0.0073 | 0.0289 | 0.800 |
| Height_Spl2 | 0.1432 | 0.0751 | 0.057 |
| Height_Spl3 | -0.5871 | 0.2852 | 0.040 |
| **W, %predmax** |  |  |  |
| Intercept1 | 7.2957 | 4.0556 | 0.072 |
| Intercept2 | 8.0272 | 4.062 | 0.048 |
| Intercept3 | 8.5781 | 4.061 | 0.035 |
| Intercept4 | 9.2673 | 4.0666 | 0.023 |
| Intercept5 | 9.7618 | 4.0692 | 0.016 |
| Intercept6 | 10.14 | 4.0784 | 0.013 |
| Intercept7 | 10.2704 | 4.0799 | 0.012 |
| Intercept8 | 10.5014 | 4.0797 | 0.010 |
| Intercept9 | 10.5654 | 4.0799 | 0.010 |
| Intercept10 | 10.7682 | 4.0817 | 0.008 |
| Intercept11 | 10.9146 | 4.0796 | 0.008 |
| W, %predmax | -0.078 | 0.009 | <0.001 |
| W, %predmax_Spl2 | 0.0951 | 0.027 | <0.001 |
| W, %predmax_Spl3 | -0.1779 | 0.0714 | 0.013 |
| Sex (male=1, female=0) | -0.25 | 0.1867 | 0.110 |
| Height | -0.0362 | 0.0254 | 0.155 |
| Height_Spl2 | 0.1621 | 0.0708 | 0.022 |
| Height_Spl3 | -0.6462 | 0.2727 | 0.018 |
| **V'O2%predmax** |  |  |  |
| Intercept1 | 10.7386 | 4.7626 | 0.024 |
| Intercept2 | 11.4284 | 4.7705 | 0.017 |
| Intercept3 | 11.9547 | 4.7705 | 0.012 |
| Intercept4 | 12.6287 | 4.7751 | 0.008 |
| Intercept5 | 13.1225 | 4.7788 | 0.006 |
| Intercept6 | 13.503 | 4.7879 | 0.005 |
| Intercept7 | 13.6343 | 4.7907 | 0.004 |
| Intercept8 | 13.8679 | 4.793 | 0.004 |
| Intercept9 | 13.9326 | 4.794 | 0.004 |
| Intercept10 | 14.1374 | 4.7957 | 0.003 |
| Intercept11 | 14.285 | 4.7947 | 0.003 |
| V'O2%predmax | -0.0921 | 0.0108 | <0.001 |
| V'O2%predmax_Spl2 | 0.1179 | 0.0311 | <0.001 |
| V'O2%predmax_Spl3 | -0.2403 | 0.0865 | 0.006 |
| Sex (male=1, female=0) | -0.3352 | 0.1895 | 0.067 |
| Height | -0.0486 | 0.0298 | 0.103 |
| Height_Spl2 | 0.1729 | 0.0779 | 0.026 |
| Height_Spl3 | -0.6627 | 0.2951 | 0.025 |

**Table S4.** Fit of the normative reference equations in the development (CanCOLD) sample, in terms of average absolute diferrence (observed-predicted, %) in probabilities for each leg discomfort (Borg CR10) score in males and females together.

| **Borg CR10 score** | **Normative reference equation** | | | |
| --- | --- | --- | --- | --- |
|  | **W, watts** | **V’O**_2_, L/min | **W, %predmax** | **V’O**_2_, %predmax |
| ³0.5 | 1.71 ± 5.57 | 1.78 ± 4.87 | 1.67 ± 6.07 | -1.28 ± 5.30 |
| ³1 | 0.30 ± 8.45 | 0.32 ± 8.29 | 0.41 ± 8.60 | -3.47 ± 7.18 |
| ³2 | -0.06 ± 11.47 | -0.02 ± 10.63 | 0.10 ± 11.54 | -4.29 ± 8.88 |
| ³3 | 3.67 ± 14.71 | 3.80 ± 14.46 | 3.84 ± 14.99 | -0.72 ± 10.76 |
| ³4 | -0.07 ± 13.89 | 0.17 ± 13.53 | 0.09 ± 13.96 | -4.12 ± 8.47 |
| ³5 | -3.16 ± 10.40 | -2.86 ± 10.24 | -3.02 ± 10.51 | -6.76 ± 5.66 |
| ³6 | -7.75 ± 6.79 | -7.44 ± 6.77 | -7.61 ± 7.13 | -11.16 ± 5.88 |
| ³7 | -6.90 ± 5.22 | -6.58 ± 5.81 | -6.77 ± 5.39 | -9.96 ± 5.45 |
| ³8 | -9.63 ± 5.92 | -9.32 ± 5.41 | -9.51 ± 5.82 | -12.59 ± 7.89 |
| ³9 | -8.34 ± 5.26 | -8.03 ± 4.83 | -8.22 ± 5.20 | -10.98 ± 7.07 |
| 10 | -9.65 ± 7.20 | -9.36 ± 6.63 | -9.53 ± 6.94 | -12.07 ± 9.03 |

**FIGURES**

**Figure S1.** Discrimination plots of model calibration in the development (CanCOLD) sample. Receiving Operating Characteristics (ROC) analysis of the probability of correct classification of the predicted vs. observed probabilities for each leg discomfort rating (Borg CR10). Models were evaluated based on absolute and relative (%pred_max_) power output (W) and rate of oxygen uptake (VO_2_) during symptom-limited, cycle, incremental cardiopulmonary exercise testing (CPET).


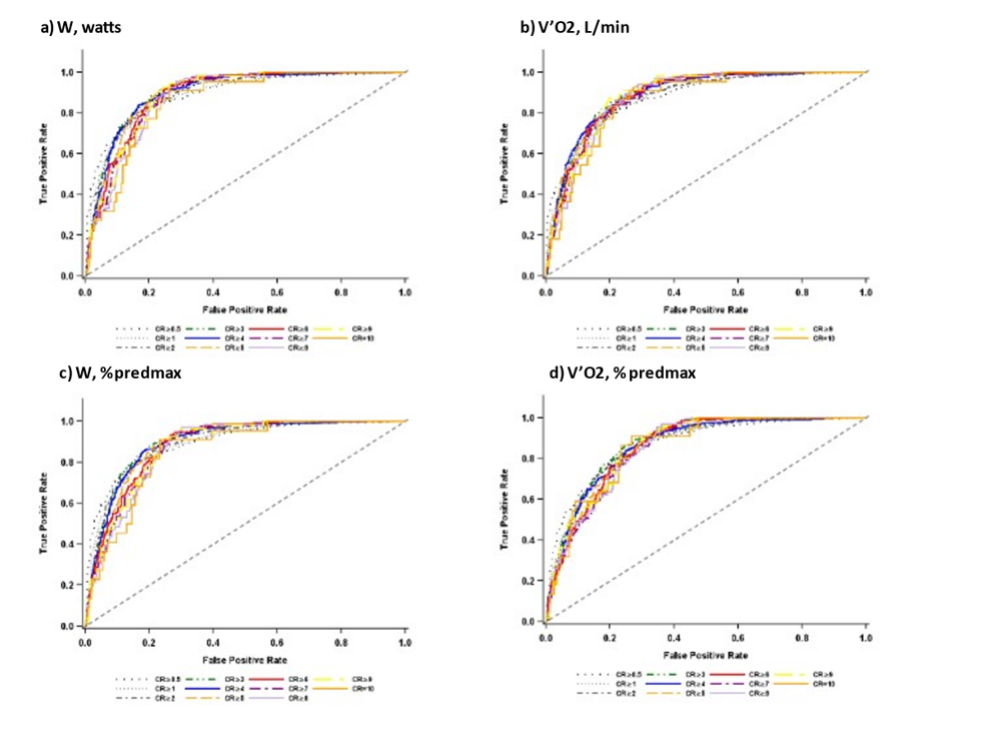


**Figure S2.** Discrimination plots of model calibration in the external validation sample of healthy adults. Receiving Operating Characteristics (ROC) analysis of the probability of correct classification of the predicted vs. observed probabilities for each leg discomfort rating (Borg CR10). Models were evaluated based on absolute and relative (%pred_max_) power output (W) and rate of oxygen uptake (VO_2_) during symptom-limited, cycle, incremental cardiopulmonary exercise testing (CPET).


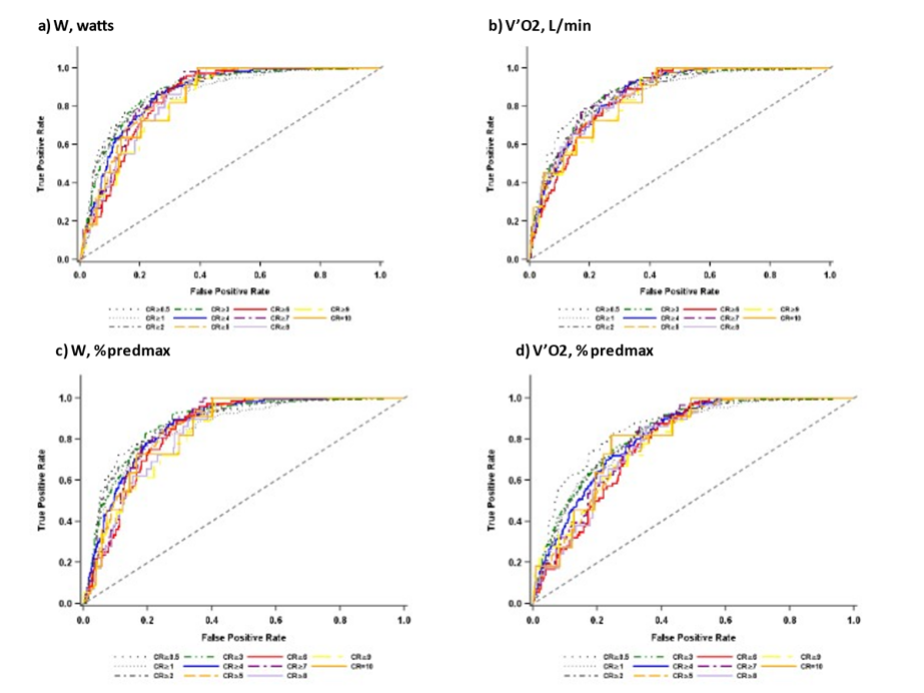


**References**

1. Lewthwaite H, Benedetti A, Stickland MK, Bourbeau J, Guenette JA, Maltais F, et al. Normative Peak Cardiopulmonary Exercise Test Responses in Canadian Adults Aged >/=40 Years. *Chest.* 2020;158(6):2532-45.

2. Quanjer PH, Stanojevic S, Cole TJ, Baur X, Hall GL, Culver BH, et al. Multi-ethnic reference values for spirometry for the 3-95-yr age range: the global lung function 2012 equations. *Eur Respir J.* 2012;40(6):1324-43.

3. Hall GL, Filipow N, Ruppel G, Okitika T, Thompson B, Kirkby J, et al. Official ERS technical standard: Global Lung Function Initiative reference values for static lung volumes in individuals of European ancestry. *Eur Respir J.* 2021;57(3).

4. Stanojevic S, Graham BL, Cooper BG, Thompson BR, Carter KW, Francis RW, et al. Official ERS technical standards: Global Lung Function Initiative reference values for the carbon monoxide transfer factor for Caucasians. *Eur Respir J.* 2017;50(3).
